# Strengthening the evidence base for decisions on public health and social measures

**DOI:** 10.2471/BLT.21.287054

**Published:** 2021-09-01

**Authors:** Delia Enria, Zijian Feng,, Atle Fretheim, Chikwe Ihekweazu, Trygve Ottersen, Anne Schuchat, Kumnuan Ungchusak,, Sylvie Briand, Victoria Haldane, Jaya Lamichhane, Ramona Ludolph, Margaux Mathis, Tim Nguyen, Nahoko Shindo

**Affiliations:** a Instituto Nacional de Enfermedades Virales Humanas Dr. Julio Maiztegui, Pergamino, Argentina.; b Chinese Center for Disease Control and Prevention, Beijing, China.; c Norwegian Institute of Public Health, Oslo, Norway.; d Nigeria Centre for Disease Control, Abuja, Nigeria.; e Atlanta, United States of America.; f Ministry of Health, Bangkok, Thailand.; g Global Infectious Hazard Preparedness Department, World Health Organization, Avenue Appia 20, 1211 Geneva 27, Switzerland.

The introduction of public health and social measures has been one of the key strategies to curb the transmission of the coronavirus disease 2019 (COVID-19). Such measures include nonpharmaceutical actions or measures such as wearing face masks, closing schools and businesses, physical distancing, restricting travel or improving air ventilation; they can be implemented by individuals, institutions, communities, and local and national governments.^1^

The use of public health and social measures has been long established in the fight against pandemic influenza.^2^^,^^3^ However, the COVID-19 pandemic has brought their implementation to an unprecedented scale and duration.

Such measures have a significant impact on the lives of individuals, on societies and the economy. Hence, evidence-informed decision-making is essential to ensure that the intervention burden of these measures does not outweigh their benefits. Thereby decisions should be based on methodologically rigorous research complemented by implementation considerations including acceptability, values and preferences. Yet, the effort researchers and policy-makers put into studying public health and social measures appears to be disproportionate to the investment in pharmaceutical trials. For example, as of 2 August 2021, 648 drug trials were reported on COVID-19 but only nine randomized studies of behavioural, environmental, social and systems interventions.^4^ Addressing this imbalance is, however, just one strategy to strengthen the evidence base on such measures. The comprehensive assessment of the benefits and harms of their implementation in different contexts also requires an increased emphasis on recognizing the insights gained from observational data.

The global evidence to date points to public health and social measures being effective in reducing the spread of COVID-19 when they are implemented in a timely, stringent and simultaneous manner with an adequate duration.^5^ A few initiatives to monitor the implementation of such measures across countries exist,^6^^–^^8^ and some studies investigate adherence to these measures across and within countries.^9^^,^^10^ However, studies disentangling the relative effects of various measures, their intervention burden and risk–benefit ratios are missing.^11^

In June 2021, the World Health Organization launched a new multiyear initiative on measuring the effectiveness and the social, health and economic impact of public health and social measures during health emergencies. The initiative aims at strengthening the global evidence base to provide actionable and evidence-informed guidance on such measures for decision-makers. Taking the larger individual and societal impact of these measures into account, the initiative will apply a multidisciplinary and multiple-methods approach promoting randomized trials and observational research.

Key expected outcomes of the first year include a global evidence review, a global research agenda and a framework to generate, monitor, compare and evaluate research and policies on such measures. As a first step, experts will assess the existing evidence, discuss ethical and methodological challenges when generating knowledge and share lessons learnt from country implementation during a technical consultation in early September 2021.

To support this initiative, the *Bulletin of the World Health Organization* calls for submission of primary studies, secondary analyses and systematic reviews on the effectiveness and broader social, health and economic impact of public health and social measures, to be reviewed on a rolling basis. Such studies are instrumental in advancing the understanding of these measures and to support decision-makers in choosing the best timing, duration, setting and combination of measures during the next public health emergency.
